# The Vaccination Fear Scale (VFS-6): Adaptation, Cross-Cultural Validation, and Invariance among Genders and Six Different Cultures, Applying Classical Test Theory (CTT) and Item Response Theory (IRT)

**DOI:** 10.3390/ejihpe14040052

**Published:** 2024-03-24

**Authors:** Olga Malas, Nada Mallah Boustani, Mirko Duradoni, Dayo Omotoso, Asiye Şengül Avşar, Anastasiia Shyroka, Giulia Colombini, Angel Blanch

**Affiliations:** 1 Department of Psychology, Sociology and Social Work, University of Lleida, Avinguda de l’Estudi General, 4, 25001 Lleida, Spain; 2 Faculty of Business and Management, Saint Joseph University, P.O. Box 17-5208 Mar Mikhael, Beirut 1104 2020, Lebanon; 3 Department of Education, Languages, Interculture, Literatures and Psychology, University of Florence, Via di San Salvi, 12, Building 26, 50135 Florence, Italy; 4 Department of Human Anatomy, Redeemer’s University, Ede 232103, Nigeria; 5 Department of Measurement and Evaluation in Education, Recep Tayyip Erdoğan University, 53100 Rize, Turkey; 6 Department of Psychology and Psychotherapy, Ukrainian Catholic University, Sventsitskogo 17, 79011 Lviv, Ukraine

**Keywords:** VFS-6, vaccination, fear, cross-cultural study, invariance, validation

## Abstract

The coronavirus disease 2019 (COVID-19) pandemic had a meaningful impact on several areas of human activity. With respect to psychological assessment, the requirements to study the fear of vaccination as a means to diminish negative behaviour towards vaccination had been reported. This study aimed to evaluate the factorial invariance of the six-item Vaccination Fear Scale (VFS-6) across individuals and cultures. To achieve this goal, a sample of university students was recruited (*n* = 2535; mean age = 20.59, *SD* = 2.04; males: 26.75%, females: 73.25%) from Spain (*n* = 388; 15.3%), Italy (*n* = 376; 14.83%), Lebanon (*n* = 487; 19.21%), Nigeria (*n* = 561; 22.13%), Turkey (*n* = 410; 16.17%), and Ukraine (*n* = 313; 12.34%). The results showed that the most appropriate factorial structure, exhibiting excellent fit indices, was a model with two correlated factors (cognitive symptoms: items 1, 2, and 4; somatic symptoms: items 3, 5, and 6) for both the total sample and individual samples from each country and language (Spanish, Italian, Arabic, English, Turkish, and Ukrainian). Notably, the VFS-6 demonstrated configural, metric, scalar, and strict invariance across sex. Regarding countries and languages, configural invariance was observed between them. Also, metric invariance was observed between Spain, Italy, and Ukraine and between Lebanon, Nigeria, and Turkey, which indicates the presence of two well-differentiated groups of countries and the possibility of inferential analysis between them. Item Response Theory analysis suggested an appropriate level of discrimination and difficulty of the test. These significant findings lay the groundwork for future investigations into vaccination fear across diverse cultural backgrounds, providing valuable insights for addressing vaccination-related concerns worldwide.

## 1. Introduction

According to the World Health Organization (WHO), vaccination prevents 2 to 3 million deaths annually, being one of the most highly cost-effective disease prevention methods [1]. Despite the compelling evidence of its benefits, fear and distrust of vaccines have led to declining vaccination rates in recent decades, emerging as one of the top ten major threats to global health [1]. Consequently, increasing vaccination rates have become a crucial topic integrated into the work plans that support the Sustainable Development Goals (SDGs) outlined in the 2030 Agenda, which has been adopted by all United Nations member states [2].

As documented in the literature, COVID-19 profoundly affected the well-being and mental health of individuals [3,4]. Although researchers unanimously recommended vaccination as one of the most efficient and cost-effective ways to curb the pandemic, people’s reluctance toward vaccination represented a critical issue in many countries [3,4,5]. Fear has been recognized as a significant factor which contributes to vaccination reluctance [6,7]. Additionally, belief in conspiracy theories, distrust in medical information, and mistrust in medicine and science have been noted [4]. In any case, fear is identified as the most powerful predictor to date concerning vaccination intention [8], particularly regarding the fear of the vaccine’s adverse effects [9,10].

Fear is commonly defined as an unpleasant emotion that is triggered in response to a stimulus perceived as potentially dangerous and can lead to protective behaviours such as avoidance [11,12]. Vaccination fear can arise from various phenomena and psychological processes related to factors such as fear of vaccine side effects and needles [13,14] and beliefs that vaccines may have no beneficial effects or could even harm individuals [13].

Research on this topic often provides the relevant information needed for the development of interventions that are aimed at reducing fear, particularly given that fear symptoms are relatively identifiable and manageable [15]. However, it is noteworthy that there is a notable lack of reliable tools available for measuring the impact of fear, which might explain the scarcity of studies in this area. However, previous studies have revealed the availability of some tools tailored to measure different aspects related to vaccination, such as worry [16,17], confidence [18], hesitancy [19], or reluctance [20,21], and other tools specifically aimed at assessing psychological barriers to vaccination [22].

Recently, the Fear of COVID-19 Scale (FoCV-19S) developed by Ahorsu et al. [23], was adapted and validated to assess fear of vaccination (6-items Vaccination Fear Scale: VFS-6) within a large Spanish sample by Malas and Tolsá [9]. Despite several attempts to adequately measure vaccination fear using unique or ad hoc items [3,13], only the VFS-6 [9] could represent the complexity of the vaccination fear construct in a valid and reliable manner by condensing the quite extensive literature related to vaccination fear [8].

Subsequently, Ochnik et al. [15] developed and validated the Coronavirus Vaccination Fear Scale (FoCVVS) concerning the German, Polish, Slovenian, and Hebrew languages, following the same methodology used by Malas and Tolsá [9] in creating the VFS-6. Despite both scales starting from the same methodology, there are certain differences between them. In the VFS-6 scale, item number 5 was excluded because it loaded almost equally on the two factors with an unacceptable loading weight (<0.50), indicating its unclear role in the factor structure. This item, however, was not removed in the Ochnik et al. [15] scale. It is worth mentioning that the VFS-6 was adapted by Malas and Tolsá [9] to measure fear of any vaccine, including the COVID-19 vaccine, thereby resulting in more generalized items. This difference may explain the variations between the two scales. 

The VFS-6 was successfully replicated by Duradoni et al. [8] with an Italian sample yielding results that are consistent with those observed in the Spanish sample. Both studies found a clear and significant association between fear of vaccination and vaccination intention, with the fear of vaccination emerging as a stronger predictor compared to hesitancy [8] or fear of the targeted disease [9]. A clear relationship has also been described between fear of vaccination and the self-report of conspiracy beliefs [24,25].

In cross-cultural research, ensuring the widespread applicability of measurement scales requires examining the equivalence of the instrument in different populations with diverse cultural backgrounds [26]. However, the development of an appropriate instrument for different cultures requires meticulous translation, content and semantic accuracy, as well as technical precision. Equally important is an accurate interpretation to avoid any construct, item-related, or method-related biases that may arise due to problematic instrument administration [27,28]. 

To facilitate a robust cross-cultural adaptation, Hinkle [26] provided a study which includes relevant validation aspects to ensure a robust fit. Also, a statistical test of the invariance of measurements between different populations is recommended. To analyse the equivalence of the measure, Lacko et al. [29] proposed a stepwise methodology based on the measurement of four different levels of invariance (configural, metric, scalar, and strict) with increasing strength. Configural invariance provides the evidence of qualitative similarity of the factor structure in different groups. Metric invariance enables the assumption that the metric remains consistent across all groups. Scalar invariance allows the assumption that the differences in item measurement between groups are due solely to differences in the latent construct being measured and not to the presence of item biases or differential item functioning. Finally, the strict invariance indicates the equality of the residual variance which implies that the measurement error is uniform across all groups.

There remains a dearth of studies regarding the invariance of the VFS-6. Duradoni et al. [8] obtained evidence of configural and metric invariance between sexes basically at the limit of metric–scalar invariance (ΔCFI = 0.005, ΔRMSEA = 0.01). On the other hand, Ochnik et al. [15] observed configurational, metric, scalar, and strict invariance between gender for the FoCVVS but only configurational invariance between countries or languages analysed. In any case, researchers in this field have emphasised that complete measurement invariance is often not met and represents too stringent a requirement for group comparison [30]. Consequently, the attainment of metric invariance has been considered sufficient to proceed with inferential analysis [31]. 

In this context, the present study seeks to translate and validate the VFS-6 in four new languages (Arabic, English, Turkish, and Ukrainian), in addition to the Spanish and Italian languages. Furthermore, this study was aimed at exploring both the structure and the invariance of the scale between the samples and their sexes, in order to verify their replicability in the different cultures that are being analysed.

## 2. Methods

### 2.1. Participants

The demographic characteristics (age, sex) and other information regarding the study participants were obtained (see Table 1). College students from Spain (*n* = 388; 15.3%), Italy (*n* = 376; 14.83%), Lebanon (*n* = 487; 19.21%), Nigeria (*n* = 561; 22.13%), Turkey (*n* = 410; 16.17%), and Ukraine (*n* = 313; 12.34%) were recruited to participate in this study. Of the pooled data set (*N* = 2535) which included students aged less than 25 years, only (Mage = 20.59, *SD* = 2.04), 73.25% were females, while 26.75% were males.

### 2.2. Procedure

This study was conducted, across the six countries, during the initial months of 2023, prior to the WHO declaration of the end of the health crisis and their recommendation to sustain seasonal vaccinations against COVID-19. University students were selected to obtain equivalent samples across countries regarding educational level, cognitive ability, and age. The recruitment process involved students from six Spanish, eight Italian, four Lebanese, five Nigerian, three Turkish, and five Ukrainian universities. The sample size per country was evaluated according to studies by Nunnally and Bernstein (p. 144 [32]), which recommended ten times the number of subjects per variable, and Tabachnick and Fidell [33], which recommended a minimum size of three hundred subjects to obtain adequate results in factor analysis. 

The instruments were administered online in the official languages of the participating countries which include Spanish, Italian, Arabic, English, Turkish, and Ukrainian. Translation and retro-translation for cross-cultural adaptation followed the guidelines proposed by Hinkle [26]. The study participants were enrolled through an online platform, using a web-link to the study questionnaire that was forwarded via e-mail to all the prospective participants, with the assistance of faculty. Prior to the administration of the questionnaire, a consent form was presented to inform the prospective participants about the research objectives and to assure them of the confidentiality of all data received. All the study participants voluntarily gave informed consent to be part of the study. The ethical approval for this study was granted by the Ethical Review Committee of the University of Lleida (Study authorization number: CERT23) as well as other institutional review boards in the participating countries.

### 2.3. Instruments

*Vaccination Fear Scale 6-items* (VFS-6): This is a six-item scale, developed by Malas and Tolsá [9], used to assess the fear of vaccination in the general Spanish population during the first wave of the COVID-19 pandemic, one month before the commencement of COVID-19 vaccination. Meanwhile, the Italian version was validated by Duradoni et al. [8] one year later, when the first round of vaccinations had finished. The scale must be adapted by introducing the name of the specific vaccine being investigated, and in the current study, it was adapted to assess fear of the COVID-19 vaccine. The scale is composed of two correlated factors, namely, cognitive symptoms (F1: items 1, 2, and 4) and somatic symptoms (F2: items 3, 5, and 6). The items were rated on a 5-point scale, from 1 (strongly disagree) to 5 (strongly agree) with scores ranging from 6 to 30. Higher scores reflect higher levels of fear related to vaccination. In the total sample, the scale revealed a Cronbach’s α of 0.87, 0.85, and 0.88 for total scale and subscales.

*Generalized Anxiety Disorder 7-item* (GAD-7): This consists of seven items measuring worry and anxiety symptoms developed by Spitzer et al. [34]. Each item is scored on a four-point Likert scale (0–3) with total scores ranging from 0 to 21. The higher the scores, the greater anxiety severity; however, scores above 10 are considered to be in the clinical range. The seven items assess different parameters including feeling nervous, anxious, or on edge; being able to stop or control worrying; worrying too much about different things; being restless; becoming easily annoyed or irritable; and feeling afraid as if something awful might happen. In the total sample, the scale yielded a Cronbach’s α of 0.94.

*Vaccination state*: A question with a dichotomous “Yes or No” response was also included to find out if the participant had been vaccinated. Studies conducted during the COVID-19 pandemic have shown that vaccinated individuals had significantly lower rates of psychological distress, anxiety, and fear compared to their unvaccinated counterparts [35]. As a result, vaccination status may influence the outcomes measured by the VFS-6, and its analysis will contribute further evidence of its validity and reliability.

*Sociodemographic information*: This includes information about the age, sex, university, and field of study of the participants. In the initial stage, age and gender were collected to create a basic profile of the sample and meet fundamental methodological standards. The inclusion/exclusion criteria involve age (less than 25 years old) and being a university student, which is why participants were also asked to provide information about the university they attend and their field of study. 

### 2.4. Statistical Analysis

As in Malas and Tolsa [9], descriptive and frequency statistics were conducted using the statistical package SPSS (version 27) to evaluate the sociodemographic characteristics, vaccination rates, scale and subscale results, and individuals’ and cultures’ differences. The factorial structure was determined in the total sample (*n* = 2535) using confirmatory factor analysis (CFA) with the maximum likelihood (ML) estimation method. Structural equation models were built using the statistical software JASP (version 0.17.2.1) with the Mplus add-on. Three solutions were analysed which include a one-factor solution, the two-factor solution proposed by Malas and Tolsá [9], and a second-order two-factor solution similar to the one proposed by Ochnik et al. [15] for the FoCVVS. To assess the fit of these models, the following indices were considered: Tucker–Lewis’s index (TLI), Comparative Fit Index (CFI), Root Mean Square Error of Approximation (RMSEA), and Standardized Mean Square Residual (SRMR). According to Hu and Bentler [36], a good fit for TLI and CFI is >0.95; for RMSEA, a good fit is <0.06; and for SRMR, a fit is considered acceptable if it is ≤0.08. 

IRT was applied to evaluate the item validity across the entire sample, with separate analyses conducted for each dimension. Employing a Graded Response Model due to our study’s utilization of a 5-point Likert scale item discrimination (α) and difficulty (β) scores were computed. According to Baker [37], α values of 0.65–1.34 = moderate; 1.35–1.69 = high; >1.70 = very high. The Item Characteristic Curve (ICC) was also examined.

To assess the construct validity of the scale, we calculated the reliability (Cronbach’s α and McDonald’s ω) for the total scale and its subscales, with α-values > 0.80 and ω-values > 0.75 being preferable [38]. Convergent validity was evaluated using the average variance extracted (AVE) with value ≥ 0.50 being preferable [38]. Divergent validity was examined based on the correlation between VFS-6, F1, and F2 (Spearman’s rho) and the heterotrait-monotrait (HTMT) ratio of correlations with value < 0.85 being preferable [39]. To determine concurrent validity, a correlation analysis (non-parametric) was carried out on the total sample between fear of vaccination and generalized anxiety determined with the GAS-7. Additionally, as an indirect test of concurrent validity, Student’s *t* test was used to examine differences in fear of vaccination between vaccinated and unvaccinated individuals (Cohen’s d was used as a measure of effect size). A linear regression analysis was also conducted to examine the relationship between the percentage of vaccination in each country (dependent variable) and the mean vaccination fear obtained with the VFS-6 (predictor variable). Bootstrapping with 250 resampling was used to increase measurement precision, as recommended by Nevitt and Hancock [40] in cases of non-normality. 

The measurement invariance (configural, metric, scalar, and strict) across the participating countries and sex was determined using multi-group confirmatory factor analysis (MGCFA). Factorial invariance between groups is assessed by comparing the equality of parameters (structure, factor loadings, intercepts, and errors) in the measurement model [29]. Starting from the established model, the invariance constraints are applied progressively. In configurational invariance, the factors and structure are the same in all groups. To calculate the metric, scalar, and strict invariance, one must progressively equalize the factor loadings, item intersections, and measurement errors. Models with invariance constraints are compared to the unrestricted model using fit indices, with the criteria for invariance being ΔCFI ≤ 0.01 and ΔRMSEA ≤ 0.015 [41].

## 3. Results

### 3.1. Descriptive Statistics

The demographic results are presented in Table 1. Despite starting with a sample focused on university students under 25 years old, the results indicate significant differences between the samples from different countries in terms of average age, sex percentage, and academic discipline.

The descriptive statistics for vaccination percentage and VFS-6 scores are shown in Table 2. Although the mean, skewness, and kurtosis values indicate an almost normal distribution for the data from Lebanon, Nigeria, and Turkey, the Kolmogorov–Smirnov *z*-test suggests departure from normality in all cases.

Non-significant differences were found for VFS-6 (*p* > 0.05) related to sex, age, or academic discipline. Female participants scored higher than male participants in F1 (*t* = −3.79; *p* < 0.001), and those under 20 scored higher than those aged 20–22 or older than 22 (*Mean difference* = 0.539–0.588; *p* = 0.002–0.012). Moreover, social science students scored higher in F2 (*Md* = 0.494 to 1.118; *p* = 0.000 to 0.035), albeit this was not significant enough to be reflected in the mean scores obtained for VFS-6 (*p* > 0.05). The effect sizes (*η*^2^) observed for sex, age, and academic discipline on vaccination fear (VFS-6) were also non-significant (*p* > 0.05). 

Regarding countries, the post hoc test with Bonferroni adjustment indicates that there was no significant difference in the mean VFS-6 scores between Spain, Italy, and Ukraine, or between Lebanon and Turkey (*p* > 0.05), but significant differences were found in the rest of the comparison (*Md* = 1.339 to 4.943; *p* < 0.001). Although the Lebanese sample showed significant differences from the Turkish sample for F1 and F2 (*Md* = 0.223 and 0.179; *p* < 0.001), the difference was not significant when analysing the total score of the scale. The same occurred with the similarity detected for F1 between the Nigerian and Turkish samples, and the Lebanese and Ukrainian samples, or the similarity in F2 between the Nigerian and Lebanese samples. The effect size (*η*^2^) observed for vaccination fear (VFS-6) attributable to the country of origin of the sample was 11.7%.

### 3.2. Confirmatory Factor Analysis

**Figure 1:**
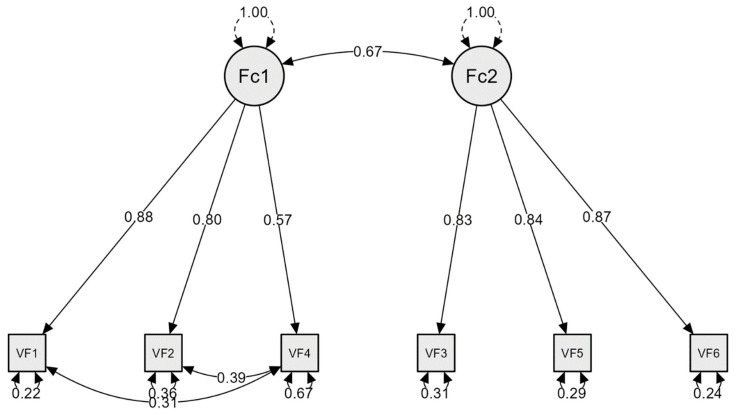
Tow correlated factor model for VFS-6. (Fc1 = Cognitive factor; Fc2 = Somatic factor; VF = VFS-6 items).

According to the results presented in Table 3, Model 1 (single factor) showed inadequate fit indices (TLI and CFI < 0.90; RMSEA > 0.10); Model 2 (two correlated factors) and Model 3 (second-order with two factors) provided acceptable fit indices (TLI and CFI > 0.95; RMSEA < 0.08). In the analysis according to countries, Model 3 consistently showed lower TLI and CFI values and higher RMSEA values. Therefore, Model 2 was selected as the most appropriate choice (see Figure 1).

### 3.3. Reliability and Validity

According to the results of this study (Table 4), the two correlated factors model of VFS-6 exhibited good internal consistency and construct reliability. Convergent validity results were satisfactory, with AVE values explaining more than 51% of the variance for both F1 and F2 in all the participating countries. The correlation analysis between VFS-6 and its factors showed values of *rho* = 0.953 and 0.748, respectively, with a value of *rho* = 0.547 between F1 and F2, indicating appropriate divergent validity between factors of the same construct. The heterotrait-monotrait ratio of correlations, which was below 0.85 in each country’s individual sample analysed further confirmed this finding. The tests conducted to analyse concurrent validity also yielded satisfactory results. The correlation data between VFS-6 and its factors concerning generalized anxiety measured by GAD-7 provided values of *rho* = 0.143, 0.117, and 0.171 for VFS-6, F1, and F2, respectively. These values indicate that VFS-6 and its subscales have low but significant correlation (*p* < 0.001) with generalized anxiety. The *t*-test for vaccination fear between vaccinated and unvaccinated individuals, along with the effect size (Cohen’s *d*), indicated that VFS-6 is capable of distinguishing between both groups. The *t*-test result is also an indirect test of the construct validity, since those with and without the relevant construct (vaccinated and unvaccinated individuals) are statistically significant. In addition, the linear regression analysis between the percentage of vaccination as the dependent variable and the average level of vaccination fear as the predictor variable indicates that vaccination fear predicts 45.8% (β = −0.169.48; *t* = 3.46; *p* = 0.025) of the vaccination response.

### 3.4. Item Response Theory (IRT) Analysis

**Figure 2:**
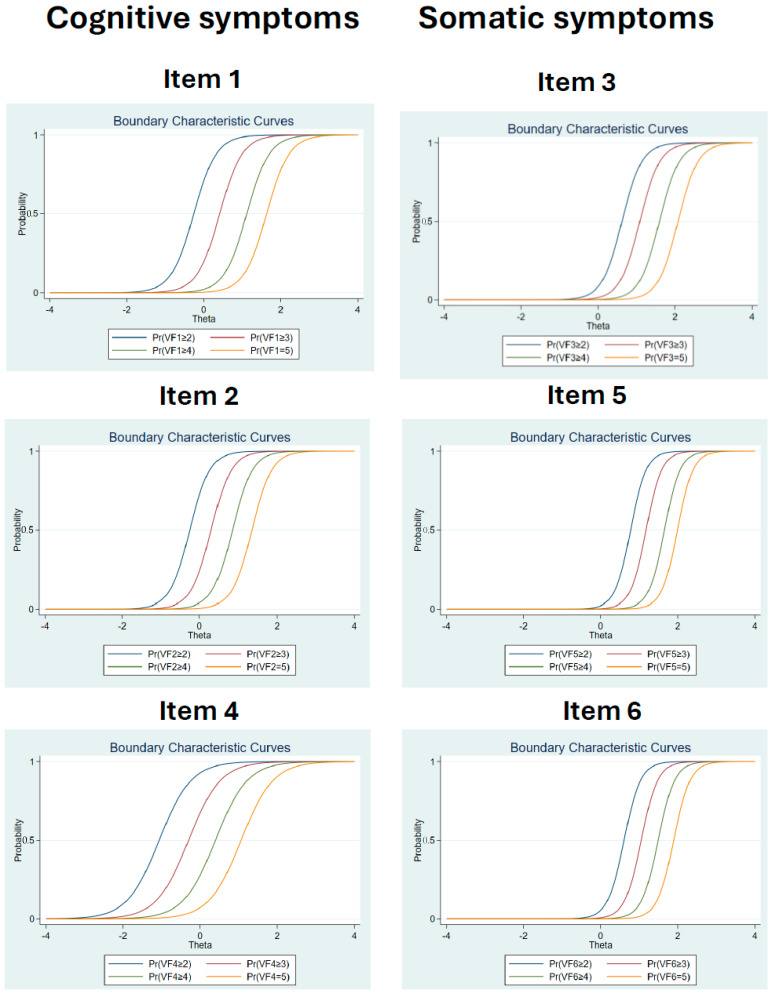
SMC Item Characteristic Curves (ICCs). The curves describe the relationship between the probability P(θ) of choosing a category option in an item.

After determining the VFS-6 scale structure through Confirmatory Factor Analysis (CFA), the Item Response Theory (IRT) was employed to assess the item validity across the entire sample. IRT analyses were conducted separately for each dimension. Given the 5-point Likert scale used in our study, a Graded Response Model was applied in the IRT analysis. Calculations of item discrimination (α) and difficulty (β) scores were performed, and the Item Characteristic Curve (ICC) was scrutinized. It is noteworthy that an α value greater than 1.0 signifies strong item discrimination, while β offers insights into the relationship between the latent trait and specific response categories for the items. The IRT results are presented in Table 5 and Figure 2 for clarity.

As indicated in Table 5, the item discrimination (α) values range from 2.40 to 3.88 for the first factor (cognitive symptoms) and from 3.85 to 4.95 for the second factor (somatic symptoms). This suggests consistently high discrimination across all items of the VFS-6. The Item Characteristic Curves (ICCs) depicted in Figure 2 exhibit S-shaped patterns, aligning with recommended standards and indicating varying levels of difficulty for items within the two VFS-6 dimensions. Specifically, items associated with the cognitive dimension are more left-shifted on the ICC graph, implying greater ease of endorsement, compared to those pertaining to the somatic dimension which showed higher difficulty. In practical terms, our findings suggested a higher likelihood of experiencing cognitive symptoms compared to somatic symptoms.

### 3.5. Measurement Invariance

The measurement invariance results by sex and country can be observed in Table 4 (M1 = configural; M2 = metric; M3 = scalar; and M4 = strict). Regarding sex, the model demonstrated acceptable fit for both male and female samples. Moreover, the model exhibited acceptable fit for M1, M2, M3, and M4, suggesting support for strict measurement invariance. Similarly, for country and language, the model demonstrated acceptable fit across all groups. However, as can be seen in Table 4, in the country/country invariance analysis, related to all countries, the model only showed configural invariance. Configural and metric invariance was observed between Spain, Italy, and Ukraine, between Lebanon, Nigeria, and Turkey, and between Ukraine and Nigeria. In turn, Ukraine presents scalar invariance with Spain and Nigeria and Nigeria with Turkey.

## 4. Discussion

The current study aimed to evaluate the psychometric properties and measurement invariance of the VSF-6 across six countries/languages (Spanish, Italian, Arabic, English, Turkish, and Ukrainian). The main outcomes confirmed that the VFS-6 has good psychometric properties, and it is suitable for the assessment of vaccination fear in the Spanish, Italian, Lebanese, Nigerian, Turkish, and Ukrainian population using their respective official languages (Spanish, Italian, Arabic, English, Turkish, and Ukrainian).

This current study successfully confirmed the two correlated factors model (cognitive symptoms and somatic symptoms) using CFA in the total sample and replicated it across sex and for the samples from the different countries. These findings align with the findings of previous studies conducted by Malas and Tolsá [9] and Duradoni et al. [8]. Additionally, the model with second-order factors proposed by Ochnik et al. [15] also showed acceptable results. However, the fit indices were slightly less robust compared to the two correlated factors model, which led us to select the two correlated factors model as the most appropriate.

No studies have been found using IRT analysis for VFS-6, nor for similar scales, so there are no reference data. But, based on the guidelines established by Becker [37] and others such as Stavropoulos et al. [42] or Wilson [43], IRT analysis suggested an appropriate level of discrimination and difficulty of the test with the somatic dimension characterised by higher difficulty compared to the cognitive dimension. These results add great value to those obtained through the CTT, since unlike this, the IRT parameters estimate the relationships between the levels of a latent trait θ and the elements and, therefore, are independent of the sample [37,43].

The internal consistency (α > 0.80; ω > 0.75) and other validity indices were highly appropriate for the scale and its subscales, both for the total sample and by countries, confirming its convergent validity (AVE ≥ 0.50), divergent validity (HTMT < 0.85), and concurrent validity. The linear regression analysis, as previously indicated by Malas and Tolsá [9] and Duradoni et al. [8], confirms that vaccination fear is a predictor of vaccination. In this case, the percentage of explained variance was 45.8%. Sex invariance observed in this study indicates that both sexes understood the content of the VFS-6 equally. This result is consistent with the information provided by Ochnik et al. [15] but differs from those reported by Duradoni et al. [8], which only obtained configural and metric invariance. Across the participating countries, configurational invariance was obtained which suggests that the structure of the two correlated factors was the most appropriate, regardless of the country and language used.

The country/country invariance also give rise to two well-differentiated groups of countries which show configural and metric invariance between them, which, according to Hsiao and Lai [31], would allow inferential analysis between them. These comprise Spain, Italy, and Ukraine on one hand and Nigeria, Lebanon, and Turkey on the other, as well as, Ukraine and Nigeria. On the contrary, Spain and Italy, in relation to Lebanon, Nigeria, and Turkey, only present configurational invariance. Metric invariance was not observed, which implies potential differences in the meaning attributed by the two groups of countries to the underlying construct being studied. This could explain the absence of scalar invariance (scores of latent variables not comparable between groups) and consequently, strict invariance, as the latent construct is not being measured identically in the two groups of countries.

The data obtained in the analysis of invariance are consistent with those obtained in the sociodemographic analysis. When examining the mean values of fear of vaccination rates, using *t*-tests and ANOVA, no significant differences (*p* > 0.05) were found attributable to sex, age, or branch of studies. In contrast, the analysis originated two groups of countries formed by Spain, Italy, and Ukraine, and by Lebanon, Turkey, and Nigeria (F2 with Lebanon and F1 with Turkey), respectively, which did not present significant differences within the group (*p* > 0.05), but this was seen with the countries of the other group. In parallel, the study of the effect size (*η*^2^) indicates that 11.7% of the fear of vaccination is attributable to the country of origin of the sample. It, therefore, seems evident that the scale is sensitive to different cultural contexts.

As detailed in the methodology section, data collection was conducted across the six countries during the initial months of 2023, prior to the WHO declaration of the end of the health crisis and their recommendation to sustain seasonal vaccinations against COVID-19. Consequently, vaccination programs are in their final phase worldwide; it can be inferred that the pandemic phase likely has not substantially impacted the levels of fear and anxiety associated with vaccination. Previous research has suggested that vaccination plans are influenced by a combination of socioeconomic and psychological factors including belief, perception, and attitude towards health which vary between countries [44]. In this study, the countries with the lowest vaccination rates were Nigeria and Ukraine. The latter country went to war with Russia in 2022, which explains the referenced rate. As can be seen in Table 1, Nigeria presents an unusual situation. Only 37.61% of the participants declare having been vaccinated at the time this study was carried out, despite the fact that 61.50% were health sciences students. The percentage of vaccinated people is far below that of participants from other countries and, at the same time, the percentage in health sciences was far above participants from other countries, which is paradoxical. In 2021, Nigeria already had vaccines against COVID-19 [45]. Despite this, on those dates, in a survey of students (67.9% in health sciences), only 40% were in favour of getting vaccinated; 15.5% were willing to pay for the vaccine, and 37.3% were willing to recommend it to others [46]. Studies carried out at that time in Spain [24] and other European countries [47] showed similar results regarding vaccination intention. But, finally, as can be seen in Table 1, in 2023 the vaccination percentages exceeded 96%. On the contrary, in Nigeria, the results are very similar to those reported in 2021 [46]. Although Mustafa et al. [46] describe sociodemographic causes, such as age, gender, and ethnicity, the main reasons cited by students for not getting vaccinated were distrust in the government regarding vaccines (73.6%) and the need to pay for them. Students also indicated that they were willing to be vaccinated if ordered by the directors of their educational institutions. It can be assumed that this educational authority did not order vaccination, which would allow us to hypothesize a distrust of vaccines beyond the university students themselves. Hence, it is undeniable that culture will influence the emotional experience of fear, particularly with respect to the secondary processes of communication and coping. Therefore, further study is needed to assess the relationship between the fear of vaccination and country-specific social variables. This will provide further understanding of how the heterogeneity of countries is reflected in the analysis and how the scale is sensitive to cross-cultural contexts.

### Limitations and Conclusions

Although the results of this study indicate that vaccination fear can be effectively measured using VFS-6, there are several limitations that need to be addressed. Firstly, the research findings can only be generalized to university students under the age of 25. It is unclear whether the scale would also be suitable for the general population in countries other than Spain and Italy where studies have been conducted for more specific population groups. Furthermore, the data reported for Ukraine should be interpreted with caution due to the potential impact of the vaccination plan amid the onset of the war with Russia in February 2022. Additionally, the study did not include countries from the Americas or the Far East, highlighting the need for further multinational research to replicate the validity of this tool in different geographical regions of the world.

Nevertheless, the reported results constitute a robust foundation, indicating that VFS-6 is a reliable and validated instrument for measuring cognitive and somatic responses to vaccination across the participating countries and languages (Spanish, Italian, Arabic, English, Turkish, and Ukrainian). Therefore, it is possible to recommend its continued application and analysis in other cultural contexts to generalize its use in determining vaccination fear. Given that unvaccinated individuals demonstrate higher vaccination fear compared to vaccinated individuals, understanding vaccination fear can be valuable in planning campaign and awareness programs aimed at increasing vaccination rates.

## Figures and Tables

**Table 1:** Demographic characteristics of the study population (TS. Total sample, *n* = 2535; 1. Spain, *n* = 388; 2. Italy, *n* = 376; 3. Lebanon, *n* = 487; 4. Nigeria, *n* = 561; 5. Turkey, *n* = 410; 6. Ukraine, *n* = 313).

	Sample Nationality							Cross-Country Comparison
	TS	1	2	3	4	5	6	
Age								
Mean	20.60	21.37	21.78	20.25	19.95	21.53	18.69	*χ*^2^*/df* = 30.4
*SD*	2.04	2.07	1.79	1.13	2.36	1.41	1.34	*p* < 0.001
Sex (%)								
Male	26.75	20.10	21.01	41.27	31.20	20.24	19.81	*χ*^2^/*df* = 17.94
Female	73.25	79.90	78.99	58.73	68.80	79.76	80.19	*p* < 0.001
Branch (%)								
Health Sci.	28.28	25.77	23.40	20.94	61.50	3.66	21.41	*χ*^2^/*df* = 45.90
Social Sci.	32.66	37.11	10.37	51.64	17.11	54.88	23.32	*p* < 0.001
Pure Sci.	10.25	12.89	2.66	13.35	12.66	12.19	4.47	
Humanities	17.59	15.21	35.37	3.90	1.60	25.12	39.30	
Engineering	5.56	0.51	8.77	6.16	7.13	0.00	11.50	
Other	5.64	8.50	19.41	4.11	0.00	4.15	0.00	
Vaccination (%)								
Yes	76.41	96.40	96.01	87.47	37.61	92.93	58.79	*χ*^2^/*df* = 156.73
No	23.59	3.60	3.99	12.53	62.39	7.07	41.21	*p* < 0.001

*Note*: *Sci*. = Science.

**Table 2:** Descriptive statistics for VFS-6 (TS. Total sample, *n* = 2535; 1. Spain, *n* = 388; 2. Italy, *n* = 376; 3. Lebanon, *n* = 487; 4. Nigeria, *n* = 561; 5. Turkey, *n* = 410; 6. Ukraine, *n* = 313).

	Sample	Vaccination (%)	Mean (*SD*)	Skewness	Kurtosis	K-S	
						*z*	*p*
VFS-6	TS	76.40	12.09 (5.56)	0.99	0.28	7.00	0.000
	1	96.39	9.82 (4.81)	2.02	4.45	4.12	0.000
	2	96.01	9.69 (4.30)	1.69	2.64	3.92	0.000
	3	87.47	12.69 (5.50)	0.84	−0.40	3.47	0.000
	4	37.61	14.63 (5.92)	0.46	−0.50	2.31	0.000
	5	92.92	13.29 (5.48)	0.74	0.17	1.91	0.000
	6	76.41	10.61 (4.58)	1.33	1.64	2.79	0.000
F1	TS	--	7.43 (3.55)	0.53	−0.79	6.94	0.000
	1	--	6.09 (3.16)	1.02	0.13	4.19	0.000
	2	--	5.90 (2.97)	1.23	0.88	3.50	0.000
	3	--	7.16 (3.13)	0.63	0.49	3.18	0.000
	4	--	9.17 (3.63)	−0.02	−1.01	1.95	0.000
	5	--	8.55 (3.57)	0.14	−0.95	1.86	0.000
	6	--	6.79 (3.32)	0.71	−0.42	2.70	0.000
F2	TS	--	4.65 (2.77)	1.88	2.76	15.92	0.000
	1	--	3.80 (2.19)	3.42	11.88	8.68	0.000
	2	--	3.79 (1.86)	2.94	8.84	8.13	0.000
	3	--	5.53 (3.04)	1.25	0.48	4.82	0.000
	4	--	5.47 (3.26)	1.20	0.33	6.12	0.000
	5	--	4.75 (2.82)	1.87	2.95	6.18	0.000
	6	--	3.81 (1.85)	3.85	13.68	6.77	0.000

*SD* = Standard Deviation; F1 = Cognitive factor; F2 = Somatic factor; K-S = Kolmogorov–Smirnov.

**Table 3:** CFA fit statistics, reliability and validity data for VFS-6 (TS. Total sample, *n* = 2535; 1. Spain, *n* = 388; 2. Italy, *n* = 376; 3. Lebanon, *n* = 487; 4. Nigeria, *n* = 561; 5. Turkey, *n* = 410; 6. Ukraine, *n* = 313).

		χ^2^/*df*	*p*	TLI	CFI	RMSEA		RMSEA IC 90%		SRMR
Model 1	TS	153.14	<0.001	0.737	0.860	0.245		0.220–0.269		0.095
Model 2	TS	8.	<0.001	0.986	0.995	0.056		0.043–0.070		0.016
	M	3.70	<0.001	0.979	0.992	0.065		0.039–0.096		0.021
	F	5.28	<0.001	0.990	0.996	0.048		0.032–0.065		0.016
	1	3.25	0.003	0.980	0.992	0.076		0.040–0.115		0.017
	2	2.88	0.008	0.981	0.992	0.071		0.033–0.111		0.018
	3	2.13	0.046	0.990	0.996	0.048		0.006–0.085		0.013
	4	3.28	0.003	0.977	0.991	0.064		0.034–0.096		0.023
	5	2.44	0.024	0.984	0.994	0.059		0.020–0.098		0.022
	6 *	2.06	0.082	0.983	0.995	0.058		0.000–0.115		0.014
Model 3	TS	12.24	<0.001	0.983	0.994	0.062		0.047–0.077		0.016
	M	4.68	<0.001	0.974	0.991	0.074		0.045–0.105		0.021
	F	6.34	<0.001	0.988	0.996	0.054		0.037–0.072		0.016
	1	3.91	0.002	0.974	0.991	0.087		0.048–0.129		0.017
	2	3.45	0.004	0.975	0.992	0.081		0.041–0.124		0.018
	3	2.56	0.025	0.986	0.995	0.057		0.018–0.096		0.013
	4	3.94	0.001	0.970	0.990	0.072		0.041–0.107		0.023
	5	2.92	0.012	0.978	0.993	0.068		0.029–0.111		0.022
	6 *	2.75	0.041	0.972	0.994	0.075		0.014–0.138		0.014
Model 2		*α*—coefficient			*ω*—coefficient			AVE		HTMT
		VFS-6	F1	F2	VFS-6	F1	F2	F1	F2	F1-F2
	TS	0.87	0.85	0.88	0.86	0.72	0.88	0.57	0.72	0.61
	1	0.87	0.84	0.91	0.83	0.76	0.93	0.57	0.83	0.68
	2	0.87	0.89	0.87	0.88	0.80	0.89	0.66	0.73	0.62
	3	0.88	0.85	0.88	0.87	0.69	0.88	0.54	0.72	0.67
	4	0.83	0.80	0.86	0.84	0.69	0.86	0.51	0.67	0.55
	5	0.84	0.83	0.99	0.84	0.70	0.89	0.55	0.73	0.53
	6	0.84	0.868	0.81	0.88	0.84	0.76	0.66	0.60	0.61
Student’s *t*-test **		*t*	*p*	Cohen’s *d*		SE Cohen’s *d*		95% CI for Cohen’s *d*		
	VFS-6	13.49	<0.001	0.63		0.05		0.54–0.72		
	F1	17.03	<0.001	0.80		0.05		0.70–0.89		
	F2	5.505	<0.001	0.26		0.05		0.17–0.35		

Model 1 = Unifactorial; Model 2 = Two correlated factor; Model 3 = Tow second-order factor. AVE = Average variance extracted. HTMT: Heterotrait-monotrait ratio. (*) The model is obtained by also correlating item 5 with item 3 and item 6. (**) Student’s *t*-test between vaccinated and unvaccinated people.

**Table 4:** Sex and country invariance (IC 90%) for VFS-6 tow correlated factors.

		ΔCFI				ΔRMSEA		
Model Comparison	CFI	M2-M1	M3-M2	M4-M3	RMSEA	M2-M1	M3-M2	M4-M3
Sex	0.995	−0.003	−0.005	−0.003	0.053	0.007	0.006	−0.004
Country								
Spain/Italy	0.992	0.001	−0.012	−0.028	0.074	−0.013	0.027	0.030
Spain/Lebanon	0.994	−0.025	−0.018	−0.070	0.062	0.060	0.015	0.044
Spain/Nigeria	0.991	−0.015	−0.003	−0.149	0.069	0.030	−0.005	0.110
Spain/Turkey	0.993	−0.019	−0.011	−0.069	0.068	0.042	0.009	0.050
Spain/Ukraine	0.987	−0.001	−0.002	−0.039	0.089	−0.007	−0.005	0.044
Italy/Lebanon	0.994	−0.018	−0.013	−0.069	0.059	0.046	0.011	0.051
Italy/Nigeria	0.992	−0.010	−0.006	−0.188	0.067	0.018	0.003	0.130
Italy/Turkey	0.993	−0.013	−0.027	−0.086	0.065	0.03	0.034	0.055
Italy/Ukraine	0.987	−0.001	−0.017	−0.028	0.087	−0.008	0.025	0.018
Lebanon/Nigeria	0.994	−0.002	−0.015	−0.034	0.057	−0.002	0.028	0.028
Lebanon/Turkey	0.995	−0.001	−0.027	−0.014	0.054	−0.004	0.055	0.002
Lebanon/Ukraine	0.990	−0.014	−0.020	−0.025	0.074	0.026	0.021	0.007
Nigeria/Turkey	0.992	0.001	−0.008	−0.031	0.062	−0.012	0.015	0.033
Nigeria/Ukraine	0.986	−0.007	−0.001	−0.097	0.08	0.005	−0.007	0.075
Turey/Ukraine	0.987	−0.009	−0.012	−0.031	0.081	0.013	0.009	0.017

Note: M1 = configural; M2 = metric; M3 = scalar; M4 = strict.

**Table 5:** Item Response Theory analysis.

Factor	Item	α	Z	*p*	β1	β2	β3	β4
F1	1	3.39	21.02	<0.001	−0.25	0.41	1.12	1.63
	2	3.88	18.12	<0.001	−0.24	0.30	0.84	1.35
	4	2.40	25.45	<0.001	−1.06	−0.30	0.40	1.06
F2	3	3.85	17.54	<0.001	0.62	1.09	1.59	2.00
	5	4.95	15.01	<0.001	0.77	1.18	1.65	1.99
	6	4.66	15.03	<0.001	0.60	1.05	1.49	1.89

## Data Availability

Data will be made available on request.
